# Stress and coping among unmarried pregnant university students in South Africa

**DOI:** 10.1186/s12884-021-04288-1

**Published:** 2021-12-09

**Authors:** Thandiwe Msipu Phiri, Patrick Nyamaruze, Olagoke Akintola

**Affiliations:** 1 Discipline of Psychology, School of Applied Human Sciences, University of KwaZulu-Natal, Durban, South Africa; 2 School of Public Health, Faculty of Community and Health Sciences, University of the Western Cape, Cape Town, South Africa

**Keywords:** Coping strategies, Early motherhood, Stressors, Unintended pregnancy, University students, South Africa

## Abstract

**Background:**

The improvement of maternal and child health (MCH) outcomes is an important part of the sustainable development goals (SDGs). MCH remains an important issue globally as the SDGs have not yet been achieved in most countries. Young women in universities are likely to experience unintended pregnancy due to risky sexual behaviors in tertiary institutions which is characterized by lack of condom and/or contraceptive use and coercion. Pregnant young women in an academic environment are susceptible to stressors associated with unintended pregnancy and academic demands of universities. However, very little is known about the stress and coping among young people in tertiary institutions who get pregnant during the course of their studies and choose to keep the pregnancy.

**Methods:**

Participants were purposively selected among pregnant students and those in the puerperal period at the time of the study. Semi-structured qualitative interviews were undertaken to explore the experiences of pregnancy and early motherhood, with particular focus on the various stressors experienced and possible coping strategies employed by students. The data were audio-recorded and transcribed verbatim, then analysed using thematic analysis.

**Results:**

The findings show that pregnancy and early motherhood was an experience that came with a lot of stress emanating from fear of parents’ reactions, academic pressure, financial constraints, relationship problems with male partners and experiences of social stigma. Participants used emotion-focused and problem-focused coping strategies to deal with the stressors confronting them during and after their pregnancy.

**Conclusion:**

The experiences of pregnant students are multifaceted and generally characterised by financial crisis, academic challenges, shame, strenuous relationships and transitioning into a new identity. A multipronged approach to healthcare for pregnant students that focus on comprehensive antenatal services, health education, health promotion, psychosocial interventions including academic counselling will have positive outcomes for young mothers and their children.

**Supplementary Information:**

The online version contains supplementary material available at 10.1186/s12884-021-04288-1.

## Introduction

Maternal health refers to the health of women during pregnancy, childbirth and the postpartum period. While pregnancy may often be a period of anticipation accompanied by feelings of maternal love and nurturing for a lot of women, it can also be a period of suffering, ill health and even death [1].

Maternal mortality is one of the leading causes of death among women. The World Health Organization [2] estimates that globally in 2017, approximately 810 women died daily from preventable causes related to pregnancy and childbirth. The majority of maternal deaths (94%) occur in low- and middle - income countries (LMICs); mostly in Africa among teenagers living in socio-economically disadvantaged settings [2, 3]. Sub-Saharan Africa and Southern Asia accounted for approximately 86% (254 000) of the estimated global maternal deaths in 2017 [2]. Most maternal and infant deaths occur in the puerperium which refers to the time period from childbirth to six weeks or post abortion [4, 5]. For example, in 2019, 47% of all under-five deaths occurred in the neonatal period, up from 40% in 1990 [6]. Regional disparities are evident in child survival with sub-Saharan Africa remaining the region with the highest under-5 mortality rate in the world [7].

Among the biggest challenges for maternal, new-born, and child health in sub-Saharan Africa are pregnancy and childbirth complications [8]. This is because pregnancy and the transition to motherhood is a period of biological and psychological changes that can sometimes cause severe stress for women. New mothers often experience decreased financial resources, physical exhaustion, task overload, confusion, social isolation and depressive symptoms [9]. These stressors can be aggravated in unplanned pregnancy.

Women who get pregnant unintentionally are less likely to initiate prenatal care in the first trimester, postnatal care after birth and have less optimal parenting practices [10, 11]. Other studies have shown that infants born to parents who report the pregnancy as unwanted are more likely to die during the neonatal and postneonatal periods [12]. Unplanned motherhood among young people has been linked to a plethora of negative prenatal and postpartum consequences such as alcohol abuse, heavy smoking and prenatal and postpartum depression. It has also been associated with the mothers’ mental health which could be a potential risk to infant and child health [13, 14]. Due to these reasons, unintended pregnancy is a concern from a public health perspective.

The reduction of maternal deaths now remains a universal goal to make childbearing a less risky experience for women worldwide. A new set of goals known as the sustainable development goals (SDGs) have been put in place and in particular under SDG3 the goal for maternal health is to reduce the global maternal mortality ratio to less than 70 per 100,000 live births as a specific maternal health indicator. Antenatal interventions have been shown to improve maternal and new-born outcomes through screening and management of pre-existing chronic diseases and infections such as HIV and malaria [15]. The health systems in low- and middle-income countries, particularly in sub-Saharan Africa, face considerable strain in providing adequate services for MCH. Gaps in provision of adequate services make it difficult to track intervention coverage to reduce maternal and child mortality ratios while responding to the growing demand for services [16]. Maternal mortality ratios are a reflection of the overall effectiveness of health systems. The health systems in most low- and middle-income countries face several technical and financial difficulties and are further characterised by a shortage of skilled personnel [2].

Sexual activity in universities and tertiary institutions in general is common all over the world [17, 18]. The university context plays a role in sexual initiation among students and sexual activity in general [17]. The freedom from parental supervision means students are free to explore the university lifestyle and some engage in sex to conform to the university sexual culture while others do it for the excitement [19]. Sexual behavior in institutions of higher learning is risky as it is usually unplanned and therefore unprotected hence posing serious reproductive health risks [20]. These risks are sometimes heightened by heavy consumption of alcohol or binge drinking, activities that normally characterize the university environment [21, 22]. Because of the risky sexual behavior among students, young women in institutions of higher learning have a high risk of unintended pregnancy [22, 23].

A study in the US utilizing secondary data from unmarried undergraduate students showed that, unintended pregnancy can lead to emotional, social, or financial difficulties that may inhibit higher educational progress [24]. Given the risks associated with pregnancy and delivery, and the stressors associated with unintended pregnancy among young people, it seems reasonable to surmise that students who choose to keep their pregnancy may be exposed to high levels of stress. Previous research among pregnant young people has focused mainly on high school students and young people who are out-of-school [25, 26]. However, very little is known about stress and coping among young people in tertiary institutions who get pregnant during the course of their studies and choose to keep the pregnancy.

The transactional model of stress and coping espoused by Folkman and Lazarus [27] enunciates how people behave when under stress. According to the authors, stress is the relationship between an individual and the environment that is appraised by the person as exceeding his or her resources and with negative implications for well-being. Coping refers to constantly changing individuals’ behavioural and psychological efforts to manage internal and/or external stressors perceived to exceed their personal resources [27]. Several coping strategies have been identified by Lazarus and Folkman: coping could be problem-focused referring to efforts to do something active to alleviate stressful circumstances, or emotion-focused, which refers to efforts to regulate the emotional consequences of stressful or potentially stressful life events. While coping with stressful events, individuals carry out cognitive appraisal of the event. This is a process of evaluation that reflects the individual’s subjective interpretation of the event [27]. First, individuals carry out primary appraisal to evaluate whether the stressful event is potentially beneficial or harmful. Next, they carry out secondary appraisal to evaluate what, if anything, can be done to prevent harm or improve the prospects for benefit [28]. Both processes - cognitive appraisal and coping – serve as mediator variables between stressful events and well-being. Young pregnant women may experience an array of stressors resulting from unintended pregnancy and the transactional model of stress and coping may be useful in explaining the ways in which these young women cope, usually by examining the balance of situational demands and perceived resources. Therefore, the aim of this study is to explore the factors influencing stress and coping among pregnant students at the University of KwaZulu-Natal who get pregnant during the course of their studies and choose to keep the pregnancy.

### Objective

We sought to achieve one objective in the study:

1. To understand the stressors experienced by unmarried pregnant students in tertiary institutions during pregnancy and the coping strategies utilised by them to deal with these stressors.

## Methods

### Study design

The qualitative research design was seen as best suited to answer the research question in this study. Qualitative research methods enable researchers to get an insider’s view of participants and their experiences [29]. The choice of a qualitative research design was primarily determined by the type of questions the study sought to address, that is, to gain a deep understanding of the experience of stress and coping among unmarried pregnant students. This design was appropriate as it fostered maximum co-operation and closeness between the interviewer and the participants and enabled them to narrate their experiences about pregnancy within the University.

We employed Braun and Clarke’s [30] thematic analysis as our methodological orientation for the study. Braun & Clarke argue that thematic analysis is an analytic tradition in its own right [30]. Thematic analysis offers a flexible and useful research tool that can provide a rich and detailed but complex account of data. It is well suited to this study because of its flexibility and because it lends itself to different theoretical frameworks [30].

### Participants

Participants in the study were all female students at the Howard College Campus of the University of KwaZulu-Natal in Durban, South Africa. Participants were recruited if they met a set of inclusion criteria which included that they: were currently registered as students at the University of KwaZulu-Natal; were currently pregnant or in the puerperal period (less than six weeks post childbirth) at the time of the study; self-identified as being single or unmarried; and were willing to answer questions regarding their pregnancy experiences.

Because issues relating to pregnancy among young people are sensitive and therefore not openly discussed, it was not easy to recruit participants for the study. Therefore, we used the snowball sampling technique in this study. This is a type of purposive sampling that allowed participants to recommend others in their circle who meet the inclusion criteria and were willing to participate in the study [31].

The recruitment of participants and the interviews were conducted by the first author (TMP). At the outset, she approached three young pregnant women who stayed in the same residence as her. She (TMP) then discussed about the study with the young women and invited them to participate in the study. Thereafter, she requested them to identify and recommend other students who were pregnant. Next, she (TMP) approached and invited those young women to participate in the study. While most of the potential participants were contacted on their cell phones and a meeting time was scheduled with them, a handful of participants were approached directly in their rooms on the campus. The interviewer was a Master’s student and a young married woman who had a pregnancy and delivered a baby while studying for her Master’s degree. She had received training on qualitative interviewing as part of her Master’s degree and additional training from the third author (OA). She conducted all the interviews drawing on her own pregnancy experiences to recruit and interact with the participants. The rationale for that was that some of the participants might be ashamed to express themselves when being interviewed by the opposite sex as demonstrated by a previous study which showed that the gender of the interviewer can substantially affect the response rate in data collection [32].

More than 30 students were invited to participate in the study and 24 were selected to participate in the study with the others excluded mainly because they were past the puerperal period and two mentioned that they were not comfortable to discuss their pregnancy experiences. Current pregnancy and puerperal period were considered because we sought to explore pregnancy experiences.

### Data collection

Ethical approval for the study was obtained from the University of KwaZulu Natal Humanities and Social Sciences Research Ethics Committee in Durban (HSS/0584/015M). In-depth interviews were conducted using an interview schedule which contained open-ended questions. The interview schedule was designed to suit the context of the study setting and to encourage pregnant students to discuss their experiences. The schedule was also reviewed and approved by the third author (OA) who was the research supervisor. The questions contained in the interview schedule included the socio-demographic variables of participants and their partners, the antecedents of the pregnancy, the challenges of pregnancy and ways of coping with the challenges.

The interviews were conducted in a private and quiet seminar room within the university. We made certain that there were no by-standers or non-participants present during the interviews. This gave participants an atmosphere of safety and comfort in which they could talk about their personal experiences considering the sensitive nature of the topic. It also helped in eliminating potential bias that might arise from participants’ reluctance to speak freely. Each interview lasted approximately 40 minutes and no repeat interviews were carried out because none of the participants was willing to be interviewed twice. We reached saturation after interviewing eight participants but continued to interview the remaining participants until all the 24 participants, who agreed to participate in the study, had been interviewed. This was done in order to include a wide range of information-rich participants that represent a wide range of experiences and to improve the rigour of the study [29]. The open-ended questions provided the participants with an opportunity to interact freely and to discuss their pregnancy experiences in detail. The interviewer (TMP) made field notes during the interview and then summarized these following the interview. The field notes were thereafter used to develop analytical memos. Written informed consent was sought and obtained from all the participants before the commencement of interviews. Permission to audio record the interviews was sought and obtained from participants prior to the interviews.

As stated earlier, the use of an interviewer who had a pregnancy experience similar to that of the students helped by making the participants feel relaxed and open up by sharing their pregnancy experiences freely. Although the interviewer received training in order to minimize bias that might occur during interviews, one cannot totally exclude the fact that some bias may have been introduced as a result of the interviewer’s previous pregnancy experience.

### Data Analysis

The audio-recorded data was transcribed by the first author (TMP). The anonymity of the participants was maintained by assigning a pseudonym to each. Consistent with the analytic tradition we employed for the study, we chose thematic analysis as the method of analysis for the data. Thematic analysis allowed us the opportunity to conduct iterations through careful reading and re-reading of the data in order to discover underlying meanings and patterns and to produce a detailed account of the phenomenon [30, 33, 34].

The first author (TMP) performed the initial round of coding following the six steps described by Braun & Clark [30]. The first step in the process was familiarization with the data by reading and re-reading the transcripts to make summaries. Secondly, she (TMP) generated initial codes and this step was followed by identifying the emerging themes, which is the third step. Fourthly, TMP reviewed potential themes, particularly checking for inconsistencies and whether the themes overlapped. After that, the themes were defined and named. Lastly, a report was produced using quotations of what the participants said to illustrate the themes [30].

It is important to note that this process was not straightforward as presented but rather was iterative. To mitigate researcher bias, the initial coding was then reviewed and modified by the third author (OA) after which the second author (PN) conducted a general review of the themes. All the authors revised, finalized and agreed upon the themes jointly.

### Findings

In total, twenty-four unmarried students participated in the study (Table 1). Among these, twenty-one were pregnant and three were in the puerperal period.

**Table 1:** Socio-demographic details of participants (n = 24)

Participant pseudonyms	Age	Gestational stage	Number of previous pregnancies
Mbali	20	Seven months pregnant	None
Lindiwe	23	Eight months pregnant	None
Busi	19	Four months pregnant	None
Samantha	19	Three months pregnant	None
Nomthandazo	19	Four weeks puerperium	None
Bongiwe	21	Three months pregnant	None
Bella	26	Six months pregnant	One
Kholeka	21	Eight months pregnant	None
Zevile	23	Five months pregnant	None
Rachael	22	Five months pregnant	None
Thuli	21	Eight months pregnant	None
Njabulo	24	Six months pregnant	None
Khethiwe	24	Four months pregnant	One
Minenhle	20	Three weeks puerperium	None
Barbara	21	seven months pregnant	None
Khanyisile	22	nine months pregnant	One
Thandeka	20	Four months pregnant	None
Lerato	21	Five weeks puerperium	None
Zinhle	23	Six months pregnant	None
Thuli	20	Three months pregnant	None
Liyana	26	Seven months pregnant	One
Siyanda	19	Four months pregnant	None
Nandi	23	Seven months pregnant	None
Lindiwe	22	Three months pregnant	None

Four of the participants had one previous pregnancy while it was the first pregnancy for the remaining twenty participants (Table 2). The findings are organized into six main themes identified from the analysis of the data: Parents’ knowledge of pregnancy and reactions; coping with the academic load; financial constraints during and after pregnancy; relationship problems with male partners; experiences of social stigma; and unexpected pregnancy experiences and adjustment.

**Table 2:** Summary of the socio-demographic details of participants

Characteristics		Number of participants (Percentage)
**Age of participants**	15-19 20-24 25-30	4 (16.7%) 18 (75%) 2 (8.3)
**Year of study**	1^st^ year 2^nd^ year 3^rd^ year Honours	7 (29%) 9 (37.5%) 5 (21%) 3 (12.5%)
**Gestational stage**	1^st^ month-birth Birth-6 weeks puerperium	21 (87.5%) 3 (12.5%)
**Number of previous pregnancies**	None 1	20 (83%) 4 (17%)

### Parents’ knowledge of pregnancy and reactions

For most of the participants, having an unintended pregnancy was a stressor because of the fear of how their parents would react when they got to know of the pregnancy. This led most of the participants to employ emotion-focused coping strategies. Denial and avoidance were major emotion-focused coping strategies employed by participants and this entails living in denial and avoiding their parents for long periods of time till it was no longer possible to continue to do so. Yet these strategies, at the same time, constituted stressors.

It’s been very difficult because as am talking to you, my parents don’t know about it [the pregnancy]. They are so strict, especially my father. He is so strict. He’s staying at Joburg [city of Johannesburg], [he’s] working there. I am pretty sure that he is going to cut me off [stop paying for her studies] because of the pregnancy. I know because this has happened to my sisters. That’s what he does. I’m stressed but am trying to control it because it’s not healthy for the baby [Mbali]

For most of the participants, the use of these coping strategies resulted in the late initiation of antenatal care.

Having to hide the pregnancy from my father whenever he was around was an issue. I had to suck in my stomach, and I started going to the clinic very late, I started going at five months and the nurses were very angry [Kholeka]

Most participants reported tension at home when they eventually disclosed their pregnancy to their parents and this was a constant stressor. In order to cope with this situation, participants reported using emotion-focused coping strategy of resignation and crying.

When my father found out that I was pregnant which was recently, he cried like a baby. My mom couldn’t talk like for a week or something. It was tough, I could not even study. I had examinations coming up in the next four days and I could not study. I would take my book, it was politics [the examination]. I would take my book and just start crying. They still can’t look at me in the eye especially my father [Samantha]

Most of the participants employed self-blame to cope with the turn of events in their homes by expressing guilt and regret over their pregnancy. They felt that they had lost their parents’ trust and the relationships they had with them had been ruined because of the pregnancy.

He [her father] kept saying to me “I trusted you so much and I still can’t believe it”. I started regretting and everything, as much as I had bonded with the baby. My father is a priest, so he is a well-respected man and people respect him. He doesn’t have much [he is not rich], he’s is just an average guy but people in our community do come to him for advice because he is a respectable man [Bongiwe]

### Pregnancy and academic work load

The physiological demands associated with pregnancy were a constant source of stress that affected participants’ academic work. Most of the participants indicated that coping with the academic workload was difficult because they were constantly feeling tired and sleepy. Our study found that severe fatigue and nausea are usually accompanied by drowsiness. It is therefore not surprising that Rachel lost concentration in her academic work which resulted in poor grades:

I dropped so hard [referring to academic score] in first semester because it was when I found out I was pregnant, and everything was so messed up. I couldn’t concentrate, I couldn’t pay my full focus [attention] on my studies, I failed. When I was trying to study, I would be [feel] sleepy, I would get a book then feel drowsy and find myself sleeping so that affected me [Rachael].

While most of the participants employed emotion-focused coping by resigning, a handful employed problem-focused coping strategies. These participants were able to take practical steps to solve their problem by drawing on their inner strength in order to attend classes as was the case with Zevile.

Walking around has been such a hassle, even going for that one seminar is so hard- sore feet, my sore back but then that only happens now in my third trimester. But during my first trimester, my first trimester was bad cause I was so sick but then I had to force myself to go to class [Zevile]

A few of the students employed problem-focused coping strategies when experiencing debilitating pregnancy symptoms. To cope with the stress associated with changing sleeping patterns coupled with severe fatigue, Thuli had to employ problem-focused strategies to help her focus on her schoolwork.

Ah sometimes I have to sleep early and not study. I don’t know this baby (is) always tiring me hey, it’s too heavy and makes me hungry. My mother always says that I sleep too much now. I have to take some treatment (for energy) every day [Thuli]

Some participants mentioned that sometimes they are torn between seeking healthcare and attending to academic work. The problem-focused coping strategy of attending antenatal clinics which was employed by some of the participants meant that they sometimes missed classes or examinations. This was common in the last trimester of pregnancy.

[Previous pregnancy]-I came here maybe I was about 6 months (pregnant). When I came on campus I usually did not attend all my lectures, (I) had to miss some of my lectures because I had to visit at the clinic to check if my baby is okay. She was born on the 3rd but she was supposed to be born on the 10th so I had to make it faster (opted for a caesarian section)…. [Current pregnancy]- Sometimes I have to leave early if I feel that I have a problem with the baby inside. I have to leave and go to the hospital and my appointments are usually on the day that am attending (lectures). I have to go see the doctor sometimes [Bella]

Being unable to attend classes and write examinations caused some participants to lag behind in their academic work and this was a source of stress for them.

Last year I had one sup (supplementary examination) and I did not write it in the first semester when I was pregnant. I didn’t know that I had to come back because my baby was too small. I didn’t come back to write the sup so I have to do it this year. I have to do five modules and the sup that I missed last year. I’m really studying hard this year. I don’t sleep, I don’t sleep [Bongiwe]

### Financial constraints during and after pregnancy

Pregnancy came with its own financial demands which sometimes could not be met by the financial resources provided by parents. Lack of financial resources was a stressor that also affected the health seeking behavior of the participants. In certain instances, some participants did not have money for transportation to ANC services. They employed problem-focused coping strategies by seeking financial support from friends and school mates. While they reported receiving help on some occasions, this strategy sometimes caused more stress for the participants.

When you’re pregnant you have to go to the clinic (antenatal clinic) each and every month and they [the clinic] are going to give you a date to come. And then when you have to go to the clinic, it happens that you don’t have money because you have to travel and get a taxi, then you don’t have money. Then I have to go around at res [student’s residence] up and down, borrowing money to go to the clinic then people will be like “I don’t have money, I don’t have money”. It’s so painful [Njabulo]

The issue of insufficient financial resources seemed to result from the low financial support from parents because of their disappointment and the unmet expectations of financial support from male partners of the participants. One participant spoke about receiving less money from her parents which she also had to share with her baby.

Now I have financial constraints because what [money] I get, the baby gets half and I get the other half [Bongiwe]

When it came to hospital bills and preparations for childbirth, participants expected their partners to provide financial support. Some of the participants mentioned lack of financial support from the father of their baby during and after pregnancy which caused a lot of stress for them. One participant expressed her disappointment as she talked about the challenges she has experienced in trying to obtain financial support from the father of her child.

It’s a challenge, it’s a big challenge with finances he [father of her child] will make an issue and an issue and an issue and am like I told you I don’t work, I don’t have nothing yet [money]. So, for me that just hurts because I have realised that he just doesn’t wanna be ready and he just doesn’t care at all [Minenhle]

There was a concern from some of the participants when it came to choosing the hospital that they wanted to deliver their baby. Participants indicated that they did not have a choice because of their financial dependence on their partners and thus were unable to access the kind of medical facilities that they would have preferred.

If your partner decides that you have to go to a government hospital because he doesn’t have money to pay, there is nothing you can do so I think it’s a very devastating position to be in [Barbara]

In some cases, male partners were financially overwhelmed and did not have enough resources to assist their partners to prepare for the birth of the baby. This was mostly because, in addition to paying the medical bills, they also had to make arrangements to pay the ‘damages’ to the family of the pregnant partner as part of custom. Paying the damages is a custom whereby a man is expected to pay an agreed-upon amount of money to a woman's family if he admits to having impregnated her out of wedlock. One participant in her final week of pregnancy said:

The circumstances surrounding the pregnancy [unintended] made it hard for us to be prepared-financially he wasn’t ready, and I wasn’t ready. Then he had to start making preparations to pay at home [damages to her parents] and he pays for all the medical bills and things financially really aren’t flowing too smooth for him. [Khanyisile]

The challenges with financial resources resulted in participants not being able to buy essential items during pregnancy.

She’s [her mother] only paying school fees and buying me clothes but not a lot of clothes. I have to wear my old clothes which are now small because of the pregnancy [Khethiwe]

## Relationship problems with male partners

Young women in this study had expectations of support from their partners and unmet expectations resulted in conflicts. Some participants reported that they had initially been told by their partners to have an abortion because they did not want to have a child. In the end, the relationship did not survive, and it ended within the first few months of pregnancy.

When I was telling my boyfriend I am pregnant, he asked me to do an abortion and I was like *what the hell*? I told him no, I wasn’t going to do it, so we argued. We continued dating but at the end of the day he still dumped me [Thandeka]

For a few of the participants, constant arguments in the relationship resulted in a communication breakdown and eventually, the relationship came to an end. In cases where the relationship had ended, communication was only initiated when the participant desired certain needs to be met. Occasionally they employed emotion-focused coping strategies.

There was a time where we just used to fight and fight, there was a point he started disrespecting me and am like I can’t take this anymore. So, I decided to just cut off all communication and that’s what I did and stopped talking to him, cause even like now we don’t talk at all the only time I spoke to him is if I want something from him, if he doesn’t have it, then it’s okay [Lerato]

When my boyfriend left me (left her due to conflicts), I hated him! I would curse and send him sms [text] and threatening him, telling him if you don’t come back, you’ll never see your child and such and such [Busi]

The participants said that they experienced stress because of conflicts with their partners. They expected support from their partners and the lack of support from their partners was a source of frustration and regret. The participants coped by employing emotion-focused strategies of accepting the situation and/or crying.

I felt like he neglected me. I was so irritated and I was crying like a baby. I felt so sad and I felt like you know, I wish I was not pregnant, like at the same time I am like I love you baby [her unborn baby], but at this point in time, I wish I wasn’t pregnant [Zinhle]

A few of the participants, who were no longer in relationships with their partners, said the amount of support they received from them was very minimal and in some cases no support was given at all. Some of the participants who had already had a baby reported that the baby’s father had not come to see the baby or sent any form of material or financial support for the baby.

He has not seen the baby from day one. I would say in a way, it’s like am just a single parent and decide for him (decisions) and the only time I get to tell him is I have decided on so and so and so. At times I don’t even consider his opinion. It’s just an opinion he won’t do anything about it [Thuli]

Unintended pregnancy in unstable relationships does not only have serious implications for maternal and child health but also for a child’s social identity when paternity is denied. A few of the participants reported that sometimes their partners denied paternity of the child because they do not want to assume the financial obligations of parenthood. They reported that this was a source of stress and that they used emotion-focused coping strategies of crying and expressing regret to deal with the situation.

I got pregnant in February then towards the end of the month we broke up. So, I didn’t know like how I am going to tell the father of the baby because we already broke up. So, when I told him, he refused, he just denied my pregnancy, started calling me names, insulting (me) saying that I’ve been sleeping around. It was so painful I was crying each and every day [Liyana]

### Experiences of social stigma

We found that most young unmarried women feel a sense of shame because of their pregnancy. Several participants mentioned experiencing feelings of shame and embarrassment at school and in their communities because of their pregnancy. This was exacerbated when people would stare at them and that was considered to be a negative attitude.

Facing people, it’s been hard like you know some people stare at you like they’ve never seen a pregnant person before. There are so many girls that get pregnant on campus [Siyanda]

Society was also said to have double standards when it came to how they judged pregnant young women. People were said to show a different, more positive attitude when they noticed the presence of a male partner as compared to when a pregnant young woman walked alone.

Walking around campus is a problem now with all the stares. It angers me and I think it’s so hypocritical in a way because their stare types change when am alone it’s like “you fell pregnant and you’re still studying” and then when my boyfriend is with me, the stare type changes all the way to “oh they love each other, how sweet” [Khanyisile]

Because of the negative attitudes from people around them and the fear of stigma, some participants said they experienced isolation and that they had lost some friends in the process. One participant talks about how certain friends distanced themselves from her and she also felt she was different from her peers and hence isolated herself. This was initially accompanied by feelings of neglect, but she later came to accept it because she realized she was different from them.

Some of my friends ignore me due to the pregnancy because they don’t wanna walk with me to school and I also see myself as totally different from them. At first, I felt so neglected now I have accepted that am pregnant and things won’t be the same. I usually walk alone. Some friends will support me, some will not [Siyanda]

Some participants coped with the fear of social stigma by delaying ANC because they did not want to be seen by members of the community at antenatal clinics.

I was afraid that my neighbours will see me going to the clinic especially to the maternity department and they will start gossiping about me. At first, I was like eish they gonna say bad comments, you know how people especially townships [Zevile]

These findings show that the socio-cultural context of pregnancy among unmarried young women still plays a role in the discourses that society creates about early motherhood.

### Unexpected pregnancy experiences and adjustments

A handful of the participants said they had a negative experience of pregnancy, which was different from what they were expecting. The participants said that, before becoming pregnant, they had expectations of getting special treatment, relaxation and being excused from certain things but found out that it was not always the case. They still had to meet their obligations at home and at school.

I thought it was lovely [pregnancy], I thought oh you get to be treated like a queen. I never really thought that unfortunately you need to toughen up, you need to pass your exams, you need to go to the clinic on your own, come home, you need to cook, you need to clean, you need to wash [Nandi].

One participant said she only realized after getting pregnant that pregnancy was not just a period that came and went away. She realized that pregnancy was the first step into motherhood, a lifelong role and responsibility that she found was not easy to accept.

I thought it was not something big, just the thought of having a baby, nothing much, that you have to stop certain things for that particular period of time when you’re pregnant. But when I became pregnant, it was different, like it changes the rest of your life. It’s not like that period only. It changes the rest of your life now you’ll have someone in your life, not just someone, but someone in a different way- someone who belongs to you, your responsibility, so that is a lifelong process and it’s not easy to accept [Lindiwe]

Some participants reported that pregnancy had caused a lot of changes in their lifestyle which they had to adjust to, and this proved to be difficult given their age and social context because they had to adapt to a new lifestyle.

So far being pregnant at a young age is not an easy thing cause we as youngsters we like partying, drinking, wearing short things so when you are pregnant when you are young, life tends to be not like it was before. Right now I have to wear something long, comfortable for the baby and also for me, the things I eat, I don’t have to go to parties anymore [Njabulo].

## Discussion

The study employs the stress and coping theory of Folkman and Lazarus [27] to provide insight into unmarried female University students’ experiences of pregnancy and early motherhood, with particular focus on the various stressors experienced and coping strategies employed. Pregnancy seemed to be a potentially good experience, in circumstances where an individual was in a stable relationship, independent and financially stable. However, for most of the participants, pregnancy was an experience that came with a lot of stress. The stressors encountered by each individual were at times unique to their different situations in their romantic relationships, at home, in the community and at school. According to Folkman and Lazarus’ theory of stress and coping [27] people use both emotion-focused and problem-focused coping strategies in almost every stressful event. Participants in our study used both strategies to deal with the stressors confronting them at various stages during their pregnancy.

One of the more commonly reported stressors among the participants was the fear of negative reactions from parents after informing them of their pregnancy. The experience of sharing the news of pregnancy with parents was stressful. This is because participants carried out both primary and secondary appraisals of their circumstances and feared being reprimanded by their parents and of disappointing their parents. Even though premarital and unplanned pregnancies are not uncommon in South Africa, parents do not approve of this practice. In a previous study, parents' potential reactions to the news of adolescent pregnancy ranged from sadness and annoyance to anger and abuse [35]. In a bid to avoid negative reactions from their parents, the young women employed emotion-focused coping strategies of fear, denial, and concealment, which were not only ineffective but also led to other problems. They concealed their pregnancy in hopes of avoiding the wrath of their parents. Unfortunately, this did not prevent them from incurring the wrath of their parents when they eventually broke the news of their pregnancy. Instead, the delay in breaking the news resulted in the late initiation of prenatal care [36]. Late initiation of prenatal care may lead to poorer health outcomes for the baby and mother, such as prenatal complications, maternal and infants' morbidity and mortality [37], which negatively impacts on goals of achieving SDG3.

Our findings demonstrate that pregnant students struggled to concentrate and overcome the pressure associated with academic load. Previous studies that show disruption to education and learning have focused on high school students. For example, a study by Stoner and colleagues among girls enrolled in high school showed that pregnancy is a major cause of school dropouts among female students [25]. All the participants in our study were currently registered as university students during their pregnancies. This entailed attendance of lectures, submission of assignments, and writing tests and exams. Because of their pregnancy, participants reported experiencing stress with respect to fulfilling their academic obligations. This study contributes to knowledge on the impact that pregnancy may have among female university students who are already burdened with academic pressures and now had to confront pregnancy-related stressors. In addition to fulfilling their academic demands, the participants struggled with physical exhaustion, fatigue, nausea and morning sickness in the first trimester of their pregnancy [38]. It was difficult for the young women to study or keep up with their academic load.

It is noteworthy that some of the participants described engaging in two types of coping strategies to deal with the stressors relating to academic load. While most of the participants used emotion-focused coping by resigning, a few employed problem-focused coping by taking practical steps to overcome the problem. They reported the use of internal strength to ‘force’ themselves to attend classes. This helped them in keeping up with their academic work. An important finding of our study is that it demonstrates that pregnancy and early motherhood can contribute to poor educational achievement among university students, a finding that has previously only been reported among high school learners [39]. Our findings show that this is especially the case with students who resorted to using emotion-focused coping strategies of resignation.

It was important for most of the participants to have enough financial resources during pregnancy. Being full time students, most of the participants did not have a source of income and were dependent on their parents and guardians for financial support. However, pregnancy came with its own financial demands which sometimes could not be met by the financial resources provided by parents. Similarly, a study among out-of-school adolescent mothers found that they experienced financial difficulties because of reduced financial support from their parents and partners after falling pregnant [26]. Lack of financial resources also affected health seeking behaviour as some participants did not have money for transportation to ANC services. Research from low-income countries in Africa has also shown that pregnancy and childbirth can be a great financial burden as pregnant women will need to frequently visit ANC sites which require transportation fare [40]. Most of the participants came from poor economic backgrounds, thus their parents were unable to provide financial support to their pregnant child. Since most of the participants indicated lack of financial support from both family and fathers of their unborn child, this resulted in the expecting mothers being unprepared for delivery in terms of the material needs of the mother and her baby [41].

The participants’ relationship with the father of the child was also explored in the current study. Unintended pregnancy, a common problem among young people in tertiary institutions, can have a negative impact on their romantic relationships. In a previous study among out-of-school adolescent mothers, participants described their relationship with the child’s father as complicated [26]. Young women in our study had expectations of support from their partners and unmet expectations resulted in conflict. The narratives from participants in our study revealed that the relationship with partners collapsed after falling pregnant leaving the female to take care of the pregnancy and the child on their own, whilst in some instances the partners denied paternity.

Research has shown that relationships among young unmarried partners are usually unstable and an unintended pregnancy can disrupt a young woman’s relationship with the child’s father [42, 43]. The disintegration of relationships during pregnancy was associated with lack of emotional and financial support. Some participants described their relationship with the father of the child as non-existing, with a previous study suggesting that when young mothers are no longer romantically involved with the father of the child, the risk of the father not being involved in the child’s life is great [44]. A few of the participants employed emotion-focused coping to address the stressors relating to the lack of provision of financial and emotional support by their boyfriends. They reported getting angry and sending unsavory text messages to their boyfriends, which exacerbated the already sour relationships.

Pregnancy was associated with negative attitudes including stigma from the community. Within an African setting, premarital sex and pregnancy out of wedlock is a shameful act that is shunned by the community [45]. Social stigma is usually an outcome of pregnancy among unmarried young women, particularly adolescents. Participants isolated themselves to avoid being stigmatised for having unplanned pregnancy whilst still in university. In some cases, participants mentioned losing close friends. Social stigma has been associated with negative outcomes such as social isolation and low self-esteem [46, 47]. The negative attitude from certain friends was said to be a reminder that because of their pregnant state, they were now different from their peers hence they felt the need to stay away from them. Other studies have reported similar findings where girls that have recently become mothers feel that they have experienced a change in social status due to motherhood [48].

Another important finding of this study is the realization by participants that contrary to their expectations, pregnancy was not always a pleasant experience, and involved increased responsibilities. Unintended pregnancy was associated with a change in roles and responsibilities for the young women who had to balance the responsibilities of being students on one hand whilst being expectant mothers on the other hand. A study among pregnant high school learners found that unintended pregnancy can cause high levels of stress among young people due to lack of readiness for pregnancy and parenthood [43]. Pregnancy was associated with the realization of a lifelong responsibility and a change in lifestyle including choice of clothing and diet. A study by Leese [49] among young mothers also found that they struggled with transitioning into roles of being a nurturer but realized the need to prioritize their children’s needs.

The findings reveal that young pregnant women might be dealing with a myriad of problems. Firstly, they will be dealing with challenges in their relationships on a personal and romantic level, which on its own without pregnancy is emotionally challenging. Secondly, the stigma of being pregnant whilst still a student, and the fear and anticipation of challenges associated with being a single parent will be imminent and these can be stressing. Thirdly, apparent lack of financial and emotional support from both the participants’ family and partner can be distressful. Lastly, the young women who are still enrolled in university need to balance all these challenges related to pregnancy and young motherhood with fulfilling their academic responsibilities. Young women have to deal with physical, psychological, emotional, academic and relationship challenges during pregnancy.

Since most university clinics offer free medical services to students, incorporating ANC services into the health services currently available on campus may improve accessibility to unmarried pregnant students and save them precious time seeking services in community clinics and hospitals. It may also be important to incorporate counselling services that target unmarried pregnant students into existing university counselling services. These services can be tailored to identify unmarried pregnant students and identify their pregnancy-related challenges and support needs which might be different from that of pregnant students who are married, engaged to be married or in union. Through these services unmarried pregnant students can receive prenatal counselling to help them cope with stress and transition to their new identity. These counselling services should focus on devising strategies to assist unmarried pregnant students to cope with common stressors such as disclosure of pregnancy to parents, negotiating relationships with partners and coping with academic work. Peer support, particularly from other young mothers may improve emotional outcomes of unmarried pregnant young women through forming relationships that reduce feelings of isolation and increase feelings of empowerment and capability through sharing of similar experiences [50].

Providing academic counselling services for unmarried pregnant students can help in achieving educational attainment. Unmarried pregnant students may need dedicated professionals to help them with time management skills and to offer regular guidance and support in navigating the complex challenges encountered in learning institutions so that they can complete their university programmes. The notable absence of partners and fathers to be of the unborn child points to the need to get young men involved in taking responsibility of their children. Health education in schools, for example the Life orientation course offered in South African public schools can integrate learning materials that focus on stressing the importance of young men’s involvement during and after pregnancy through providing emotional and financial support.

### Limitations of the study

This study explored the experiences (challenges) of unmarried pregnant students at the University of KwaZulu-Natal, Howard college campus. First, we used snowballing, a purposive sampling technique to recruit participants. This ensured that participants were from the same social network and as such pregnant women from other social networks may have been excluded by this technique. Second, while any unmarried, pregnant student qualified to participate in this study, only indigenous African students participated in this study. The snowball method of sampling that was used meant that the initial participants, who were indigenous African students, recommended other pregnant students in their social network, who were also indigenous African students. As a result, potential participants from other racial groups did not participate in this study. Therefore, the findings could be biased and be a representation of experiences and perceptions of pregnant indigenous African students. Future studies should include different racial groups to capture the experiences of pregnant students of all races. Third, the findings of this study applies only to pregnant students who are unmarried and does not apply to those pregnant students who are in a marriage or engaged to be married.

## Conclusion

In summary, the experience of unmarried pregnant students, as shown by the study findings reported here are multifaceted and generally characterised by financial crisis, academic challenges, shame, strenuous relationships and transitioning into a new identity. The findings enhanced our understanding of the educational and psychosocial barriers that stifle the positive development of pregnant students. Pregnancies, particularly in a context where it is unplanned, have marked financial implications for young women and the household at large. A multipronged approach to healthcare for pregnant students that focus on comprehensive antenatal services, health education, health promotion, psychosocial interventions including academic counselling will have positive outcomes for adolescent mothers and their children.

## Supplementary Information

**Additional file 1.**

## Data Availability

The data used to elicit the findings of this study are available from the corresponding author upon reasonable request.

## Abbreviations

SDGs: Sustainable development goals
LMICs: Low- and middle-income countries
ANC: Antenatal care
MCH: Maternal and child health

## References

[1] Koblinsky M, Chowdhury ME, Moran A, Ronsmans C. Maternal morbidity and disability and their consequences: neglected agenda in maternal health. J Health Popul Nutr. 2012;30(2):124-130. 16. 10.3329/jhpn.v30i2.11294 10.3329/jhpn.v30i2.11294 PMC3397324 22838155

[2] World Health Organization. Maternal mortality. Geneva: WHO. 2019. https://www.who.int/news-room/fact-sheets/detail/maternal-mortality. Accessed 20 Feb 2021.

[3] Gyesaw NY, Ankomah A (2013). Experiences of pregnancy and motherhood among teenage mothers in a suburb of Accra, Ghana: a qualitative study. Int J Womens Health..

[4] Merdad L, Ali MM (2018). Timing of maternal death: levels, trends, and ecological correlates using sibling data from 34 sub-Saharan African countries. PLoS One..

[5] Nour NM (2008). An introduction to maternal mortality. Rev Obstet Gynecol..

[6] UNICEF. Levels & Trends in Child Mortality. 2020 https://www.unicef.org/media/79371/file/UN-IGME-child-mortality-report-2020.pdf.pdf Accessed 27 May 2021.

[7] World Health Organization. Children: improving survival and well-being. Geneva: WHO. 2020. https://www.who.int/news-room/fact-sheets/detail/children-reducingmortality#:~:text=Since%201990%2C%20the%20global%20under,1%20in%2027%20in%202019. Accessed 19 May 2021.

[8] Kinney MV, Kerber KJ, Black RE, Cohen B, Nkrumah F, Coovadia H (2010). Sub-Saharan Africa's mothers, newborns, and children: where and why do they die?. PLoS Med..

[9] Uno D, Florsheim P, Uchino BN (1998). Psychosocial mechanisms underlying quality of parenting among Mexican-American and white adolescent mothers. J Youth Adolesc..

[10] Cheng D, Schwarz EB, Douglas E, Horon I (2009). Unintended pregnancy and associated maternal preconception, prenatal and postpartum behaviors. Contraception..

[11] Goto A, Yasumura S, Reich MR, Fukao A (2002). Factors associated with unintended pregnancy in Yamagata. Japan. Soc. Sci. Med..

[12] Chalasani S, Casterline JB, Koenig MA (2007). Unwanted childbearing and child survival in Bangladesh.

[13] Gipson JD, Koenig MA, Hindin MJ (2008). The effects of unintended pregnancy on infant, child, and parental health: a review of the literature. Stud Fam Plan..

[14] Joyce T, Kaestner R, Korenman S (2000). The stability of pregnancy intentions and pregnancy-related maternal behaviors. Matern Child Health J..

[15] Lassi ZS, Mansoor T, Salam RA, Das JK, Bhutta ZA. Essential pre-pregnancy and pregnancy interventions for improved maternal, newborn and child health. Reprod Health. 2014;11(1):1-9. 10.1186/1742-4755-11-S1-S2 10.1186/1742-4755-11-S1-S2 PMC4145858 25178042

[16] Requejo JH, Bryce J, Barros AJ, Berman P, Bhutta Z, Chopra M, Daelmans B, De Francisco A, Lawn J, Maliqi B, Mason E (2015). Countdown to 2015 and beyond: fulfilling the health agenda for women and children. Lancet..

[17] Akintola O, Ngubane L, Makhaba L (2012). ‘I did it for him, not for me’: An exploratory study of factors influencing sexual debut among female university students in Durban. South Africa. J Health Psychol..

[18] Hittner JB, Owens EC, Swickert RJ (2016). Influence of social settings on risky sexual behavior. SAGE Open..

[19] Petersen I, Bhagwanjee A, Makhaba L (2001). Understanding HIV transmission dynamics in a university student population in South Africa: A qualitative systemic approach. J Psychol Afr..

[20] Lewis JE, Miguez-Burbano MJ, Malow RM (2009). HIV risk behavior among college students in the United States. Coll Stud J..

[21] Cooper ML. Alcohol use and risky sexual behavior among college students and youth: evaluating the evidence. J Stud Alcohol Suppl. 2002;(14):101-17. 10.15288/jsas.2002.s14.101 10.15288/jsas.2002.s14.101 12022716

[22] Brown JL, Vanable PA (2007). Alcohol use, partner type, and risky sexual behavior among college students: Findings from an event-level study. Addict Behav..

[23] Sriprasert I, Chaovisitsaree S, Sribanditmongkhol N, Sunthornlimsiri N, Kietpeerakool C (2015). Unintended pregnancy and associated risk factors among young pregnant women. Int J Gynaecol Obstet..

[24] Buhi ER, Marhefka SL, Hoban MT (2010). The state of the union: Sexual health disparities in a national sample of US college students. J Am Coll Health..

[25] Stoner MC, Rucinski KB, Edwards JK, Selin A, Hughes JP, Wang J (2019). The relationship between school dropout and pregnancy among adolescent girls and young women in South Africa: A HPTN 068 analysis. Health Educ Behav..

[26] Govender D, Naidoo S, Taylor M (2020). “I have to provide for another life emotionally, physically and financially”: understanding pregnancy, motherhood and the future aspirations of adolescent mothers in KwaZulu-Natal South. Africa. BMC Pregnancy Childbirth..

[27] Folkman S, Lazarus RS (1984). Stress, appraisal, and coping.

[28] Folkman S, Lazarus RS, Gruen RJ, DeLongis A (1986). Appraisal, coping, health status, and psychological symptoms. J Pers Soc Psychol..

[29] Ulin PR, Robinson ET, Tolley EE (2005). Qualitative Methods in Public Health: A Field Guide for Applied Research.

[30] Braun V, Clarke V (2006). Using thematic analysis in psychology. Qual Res Psychol..

[31] Polkinghorne DE (2005). Language and meaning: Data collection in qualitative research. J Couns Psychol..

[32] Padfield M, Procter I (1996). The effect of interviewer's gender on the interviewing process: A comparative enquiry. Sociology..

[33] Babbie E (2010). Research design. The practice of social research.

[34] Rice PL, Ezzy D (1999). Qualitative research methods: A health focus.

[35] Aziato L, Hindin MJ, Maya ET, Manu A, Amuasi SA, Lawerh RM (2016). Adolescents' responses to an unintended pregnancy in Ghana: a qualitative study. J Pediatr Adolesc Gyneco..

[36] Friedman SH, Heneghan A, Rosenthal M (2009). Characteristics of women who do not seek prenatal care and implications for prevention. J Obstet Gynecol Neonatal Nurs..

[37] Kaswa R, Rupesinghe GF, Longo-Mbenza B (2018). Exploring the pregnant women's perspective of late booking of antenatal care services at Mbekweni Health Centre in Eastern Cape, South Africa. Afr J Prim Health Care Fam Med.

[38] Nkosi KB, Makhene A, Matlala S (2019). Educational challenges as experienced by pregnant student nurses at a college in Mpumalanga. Curationis..

[39] Maemeko EL, Nkengbeza D, Chokomosi TM (2018). The impact of teenage pregnancy on academic performance of grade 7 learners at a school in the Zambezi region. Open J Soc Sci..

[40] Dahab R, Sakellariou D (2020). Barriers to Accessing Maternal Care in Low Income Countries in Africa: A Systematic Review. Int J Environ Res Public Health..

[41] Scorgie F, Blaauw D, Dooms T, Coovadia A, Black V, Chersich M. “I get hungry all the time”: experiences of poverty and pregnancy in an urban healthcare setting in South Africa. Glob Health. 2015;11(1):1-12. 10.1186/s12992-015-0122-z 10.1186/s12992-015-0122-z PMC4549107 26303952

[42] Benjamin Guzzo K, Furstenberg FF (2007). Multipartnered fertility among young women with a nonmarital first birth: Prevalence and risk factors. Perspect Sex Reprod Health..

[43] East PL, Chien NC, Barber JS (2012). Adolescents' pregnancy intentions, wantedness, and regret: Cross-lagged relations with mental health and harsh parenting. J Marriage Fam..

[44] Gee CB, Rhodes JE (2003). Adolescent mothers' relationship with their children's biological fathers: Social support, social strain and relationship continuity. J Fam Psychol..

[45] Mwaba K (2000). Perceptions of teenage pregnancy among South African adolescents. Health SA Gesondheid..

[46] Wiemann CM, Rickert VI, Berenson AB, Volk RJ. Are pregnant adolescents stigmatized by pregnancy?. J Adolesc Health. 2005;36(4):352-e1-352-e7. 10.1016/j.jadohealth.2004.06.006 10.1016/j.jadohealth.2004.06.006 15780793

[47] Zwang J, Garenne M (2008). Social context of premarital fertility in rural South-Africa. Afr J Reprod Health..

[48] Zanchi M, da Costa Kerber NP, Biondi HS, da Silva MR, Gonçalves CV (2016). Teenage maternity: life’s new meaning?. J Hum Growth Dev..

[49] Leese M (2016). The bumpy road to ‘becoming’: capturing the stories that teenage mothers told about their journey into motherhood. Child Fam Soc Work..

[50] McLeish J, Redshaw M (2017). Mothers’ accounts of the impact on emotional wellbeing of organised peer support in pregnancy and early parenthood: a qualitative study. BMC Pregnancy Childbirth..

